# Drinking by amphibious fish: convergent evolution of thirst mechanisms during vertebrate terrestrialization

**DOI:** 10.1038/s41598-017-18611-4

**Published:** 2018-01-12

**Authors:** Yukitoshi Katayama, Tatsuya Sakamoto, Kazuhiro Saito, Hirotsugu Tsuchimochi, Hiroyuki Kaiya, Taro Watanabe, James T. Pearson, Yoshio Takei

**Affiliations:** 1 Laboratory of Physiology, Atmosphere and Ocean Research Institute, University of Tokyo, 5-1-5 Kashiwanoha, Kashiwa, Chiba 277-8564 Japan; 2 Ushimado Marine Institute, Faculty of Science, Okayama University, 130-17 Kashino, Setouchi, Okayama 701-4303 Japan; 3 Department of Cardiac Physiology, National Cerebral and Cardiovascular Center Research Institute, 5-7-1 Fujishirodai, Suita, Osaka 565-8565 Japan; 4 Department of Biochemistry, National Cerebral and Cardiovascular Center Research Institute, 5-7-1 Fujishirodai, Suita, Osaka 565-8565 Japan

## Abstract

Thirst aroused in the forebrain by angiotensin II (AngII) or buccal drying motivates terrestrial vertebrates to search for water, whereas aquatic fish can drink surrounding water only by reflex swallowing generated in the hindbrain. Indeed, AngII induces drinking through the hindbrain even after removal of the whole forebrain in aquatic fish. Here we show that AngII induces thirst also in the amphibious mudskipper goby without direct action on the forebrain, but through buccal drying. Intracerebroventricular injection of AngII motivated mudskippers to move into water and drink as with tetrapods. However, AngII primarily increased immunoreactive c-Fos at the hindbrain swallowing center where AngII receptors were expressed, as in other ray-finned fish, and such direct action on the forebrain was not found. Behavioural analyses showed that loss of buccal water on land by AngII-induced swallowing, by piercing holes in the opercula, or by water-absorptive gel placed in the cavity motivated mudskippers to move to water for refilling. Since sensory detection of water at the bucco-pharyngeal cavity like ‘dry mouth’ has recently been noted to regulate thirst in mammals, similar mechanisms seem to have evolved in distantly related species in order to solve osmoregulatory problems during terrestrialization.

## Introduction

**Figure 1:**
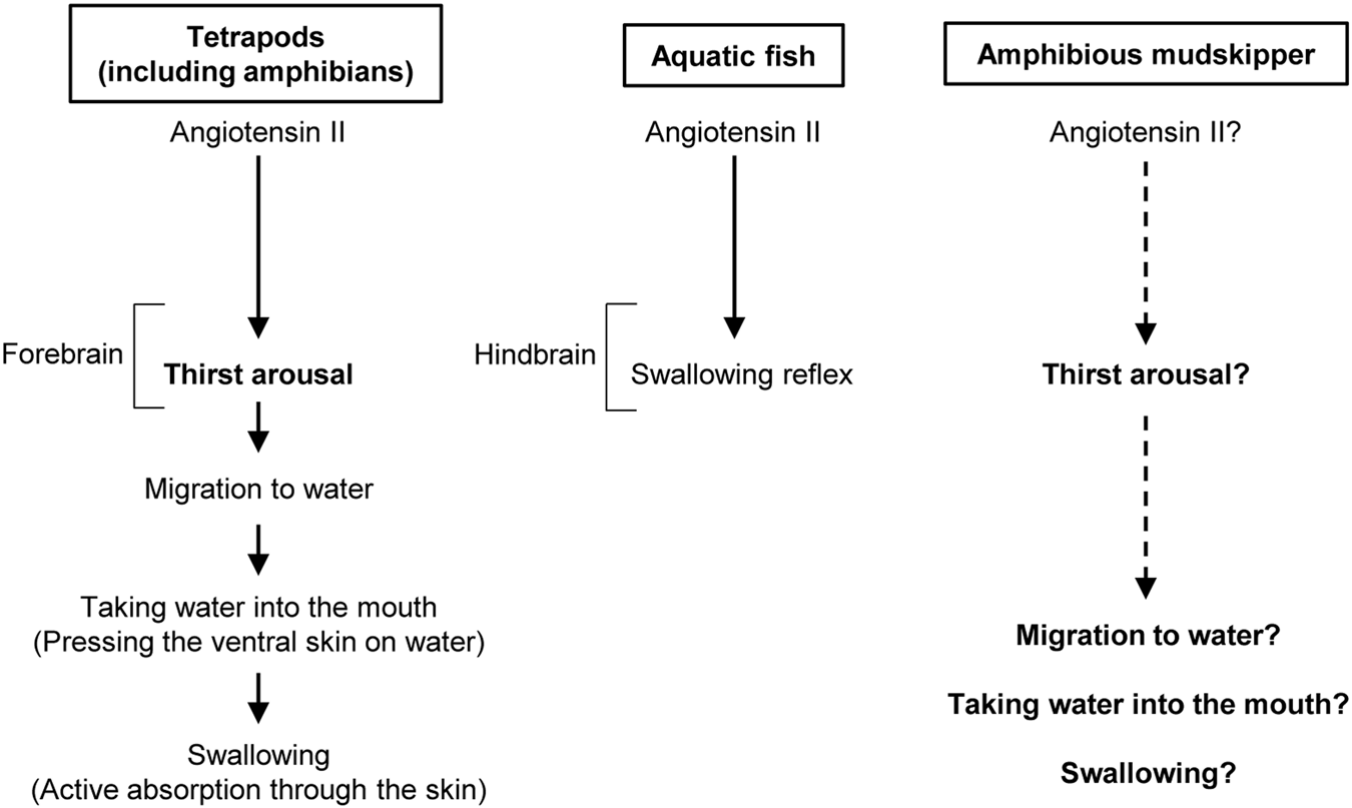
Differences in the drinking processes among habitats in vertebrates. In tetrapods, systemic angiotensin II evokes thirst to motivate a series of drinking behaviours. Fully aquatic fish can complete drinking without thirst because water available for swallowing is always present in the mouth. Mudskipper fish are amphibious gobies that spend the greater part of their lives out of water and it is intriguing to examine whether thirst followed by a series of drinking behaviours is induced by angiotensin II.

Thirst is defined as a conscious sensation of a need for water and a motivation to drink^1,2^. In terrestrial animals such as mammals, thirst is accompanied by a search for water, and its motivation or consciousness is generated in the hypothalamic area and integrated in the limbic system^3^. Angiotensin II (AngII) is the most potent dipsogenic hormone thus far known in all vertebrates, including fish (Fig. 1)^4–7^. In mammals and birds, systemic AngII has been shown to act on the sensory circumventricular organs (CVOs) in the forebrain that lack the blood-brain barrier (BBB) to induce a series of thirst-motivated behaviours^8–11^. The organum vasculosum of the lamina terminalis (OVLT) and the subfornical organ (SFO) are known as forebrain CVOs^8,9^. Although the neural basis of drinking behaviour is not fully understood in other vertebrate taxa, the occurrence of thirst has been suggested in terrestrial vertebrates including amphibians. In toads, AngII has also been shown to induce thirst at the forebrain to motivate them to water for absorption through the ventral skin, termed ‘cutaneous drinking’^12^.

In ray-finned fish that live exclusively in water, however, none of the regions in the forebrain appears to be implicated in elicitation of drinking, since our removal of the whole forebrain did not affect the drinking induced by AngII in eels^13^. Because water is always present in the mouth of aquatic fish, they can complete drinking solely by the swallowing reflex generated in the hindbrain. Indeed, we showed that AngII acted on the hindbrain CVO, area postrema (AP), to induce drinking in eels^14^. There is no evidence for thirst regulated by angiotensin’s action in the fish forebrain^15^. Thus, it has long been held that AngII-induced thirst is essential for terrestrial adaptation, and may have evolved during the terrestrialization of tetrapods (Fig. 1)^16^.

The land invasion by vertebrates in the evolutionary process occurred not only in lobe-finned fish that lead to tetrapods, but also in amphibious ray-finned fish which are independent of the tetrapod lineage^17,18^. For instance, mudskipper fish are amphibious gobies that spend the greater part of their lives out of water to feed and avoid capture by aquatic predators^19,20^. They have behavioural and physiological specializations adapted to a semi-terrestrial lifestyle^19–22^. For example, they can store water in the buccal and opercular cavities when they are on land, because their opercula are closed without ventilation of the gills. Buccal/opercular water prevents the gills from desiccation and maintains the gill function mainly for ion regulation and respiration^23,24^. Mudskippers dehydrated on mudflat likely search for water to drink like terrestrial tetrapods. Together with the recent identification of the possible OVLT in the mudskipper^25^, it is intriguing to examine whether thirst is also induced by AngII in this amphibious fish (Fig. 1).

The present study investigated the presence of thirst in the mudskipper (*Periophthalmus modestus*). We showed that AngII induces a series of drinking behaviour in the mudskipper without direct action on the forebrain but through buccal drying. We also discuss the evolution of the thirst-inducing mechanisms during the transition of vertebrates from aquatic to terrestrial habitats.

## Results

### A series of thirst-motivated behaviours is induced by AngII in the mudskipper ‘fish’

Initially, angiotensinogen cDNA was cloned from the mudskipper liver (Accession number: LC121030) to determine the amino acid sequence of AngII and to use the homologous AngII in this study (Supplementary Fig. 1a). The sequence of AngII was determined as NRVYVHPF which is identical to those of most ray-finned fish^26^.

**Figure 2:**
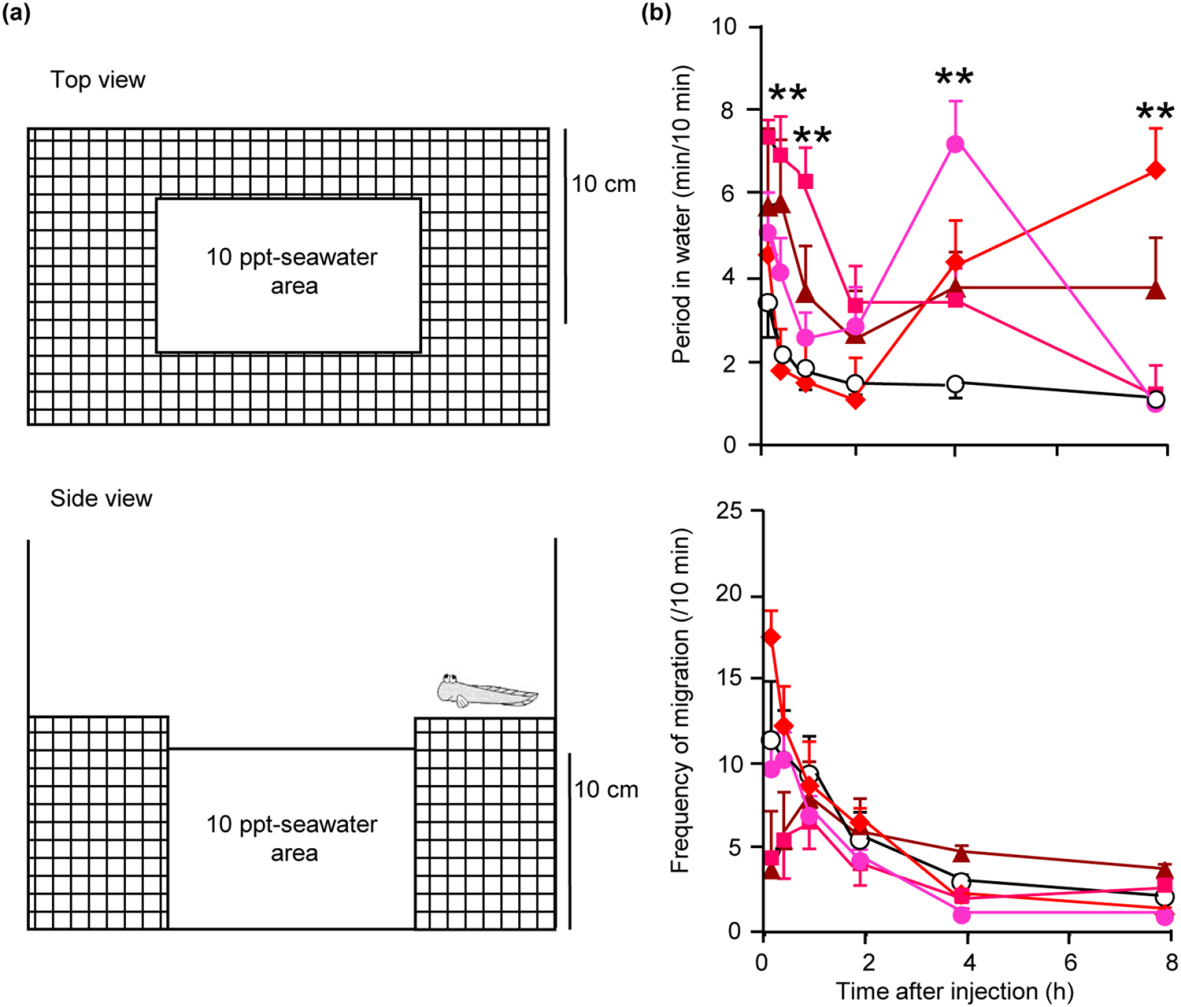
Drinking behaviour in the mudskipper induced by angiotensin II (AngII). (**a**) Schematic diagram of the experimental tank used to observe the amphibious behaviour. 10-ppt seawater is close to their natural environment and almost identical to the salinity of body fluids, and thus chosen for the subsequent experiments (Supplementary Fig. 2). (**b**) Time course changes in amphibious behaviour after injection of AngII. The period of time in water and the frequency of migration were measured after intracerebroventricular (ICV) injection with 0.1 μl/g of 3 × 10^−6^ M (, n = 6), 3 × 10^−8^ M (, n = 5), 3 × 10^−10^ M (, n = 7), 3 × 10^−12^ M (, n = 7), and vehicle (◯, n = 8) of AngII. The ICV procedure was chosen since the inconsistent behaviours were exhibited after the peripheral injection of AngII. The parameters were measured at 0.25 h (0.13–0.38 h), 0.5 h (0.25–0.75 h), 1 h (0.75–1.25 h), 2 h (1.75–2.25 h), 4 h (3.75–4.25 h), and 8 h (7.75–8.25 h) after injection. Data are shown as mean ± s.e.m.; the absence of error bars indicates a small s.e.m. Two-way repeated measures ANOVA and Dunnett’s post-hoc test were used for statistical analysis. **P < 0.01 versus controls.

Mudskippers stayed on the land area of the experimental tank (Fig. 2a) about 80% of their time during 8-h observation period when water in the tank was 10-ppt seawater (Fig. 2b, Supplementary Fig. 2). After the ICV injection of 3 × 10^−8^ M AngII, the mudskippers spent more time in water than vehicle-injected controls within the first 30-min period (Fig. 2b). The effect of 3 × 10^−8^ M AngII continued for at least an hour. The prolonged period in the water area also occurred after 4 h at 3 × 10^−6^ M and after 8 h at 3 × 10^−10^ M. There was no difference in the frequency of migration between water and land areas at any dose (Fig. 2b). The amount of water ingested also increased after AngII injection (Supplementary Fig. 3a), confirming the dipsogenic effect of AngII in this species.

### The hindbrain AP is the site of action of AngII as shown in aquatic fish

**Figure 3:**
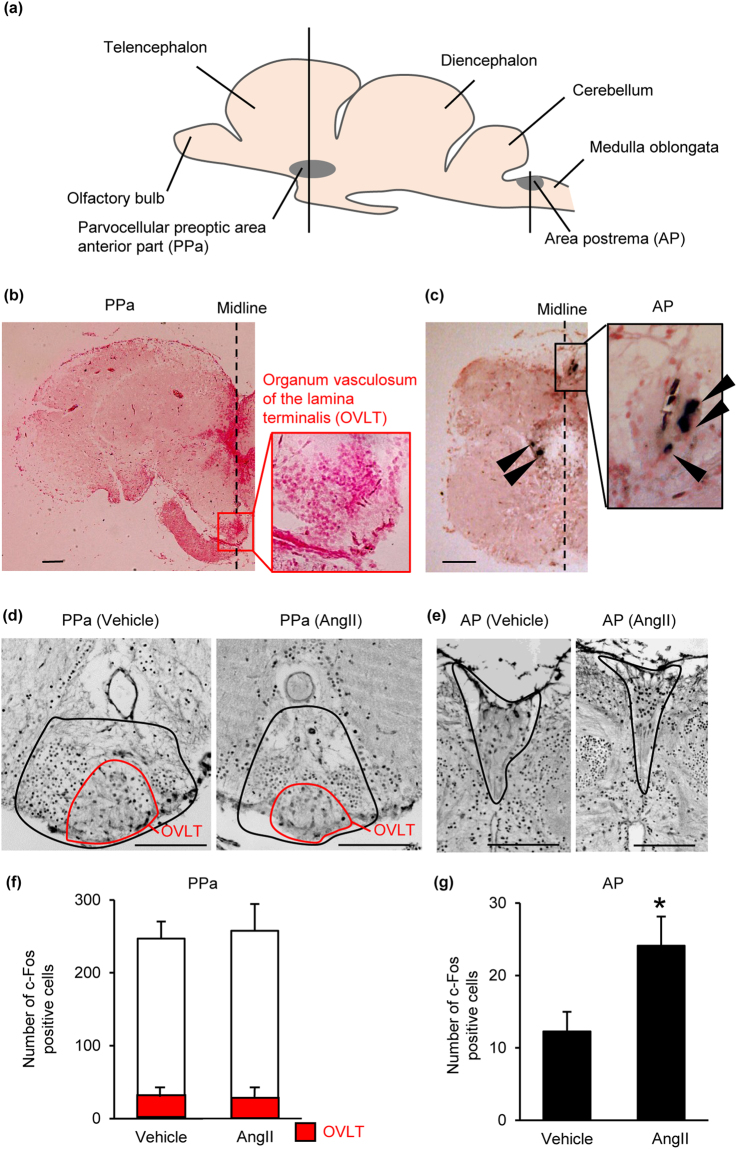
The site of action of angiotensin II (AngII) in the mudskipper brain. (**a**) Gross anatomy of the mudskipper brain at the mid-sagittal plane showing localization of the circumventricular organs (CVOs, gray-shaded regions). Vertical lines show the sites where cross sections were made. (**b**,**c**) *In situ* hybridization of angiotensin receptor type 1. Broken lines indicate the midlines and arrow heads show the positive cells. No signal was detected in the parvocellular preoptic area anterior part (PPa) including the possible organum vasculosum of the lamina terminalis (OVLT, inset). Some signals were detected in the area postrema (AP). The sections were counterstained with Nuclear Fast Red. (**d**,**e**) The c-Fos positive cells in the CVOs after intracerebroventricular injection with 0.1 μl/g of 3 × 10^−8^ M AngII or vehicle. Black lines delineate the PPa (**d**) and the AP (**e**), and red lines delineate the OVLT (**d**). (**f**,**g**) Number of c-Fos positive cells in the PPa including the OVLT (red columns) and in the AP after the injection of AngII or vehicle. Data are shown as mean ± s.e.m. (*n* = 5). *P < 0.05 with unpaired *t* test. Scale bars, 100 μm.

We examined the site of action of AngII in the mudskipper brain by the presence of its receptor and increased c-Fos immunoreactivity (Fig. 3). Localization of angiotensin type 1 receptor (AT1), the receptor for the dipsogenic action of AngII, was analyzed by *in situ* hybridization after cloning of its cDNA from the mudskipper brain (Accession number: LC171725; Supplementary Fig. 1b,c). Some AT1-positive neurons were detected in the AP, but not in the parvocellular preoptic area anterior part (PPa) including the OVLT-like structure (Fig. 3b,c). ICV injection of AngII increased the number of c-Fos immunopositive neurons only in the AP, although positive neurons were also detectable in the PPa (Fig. 3d–g).

### The loss of buccal/opercular water induces migration to water

**Figure 4:**
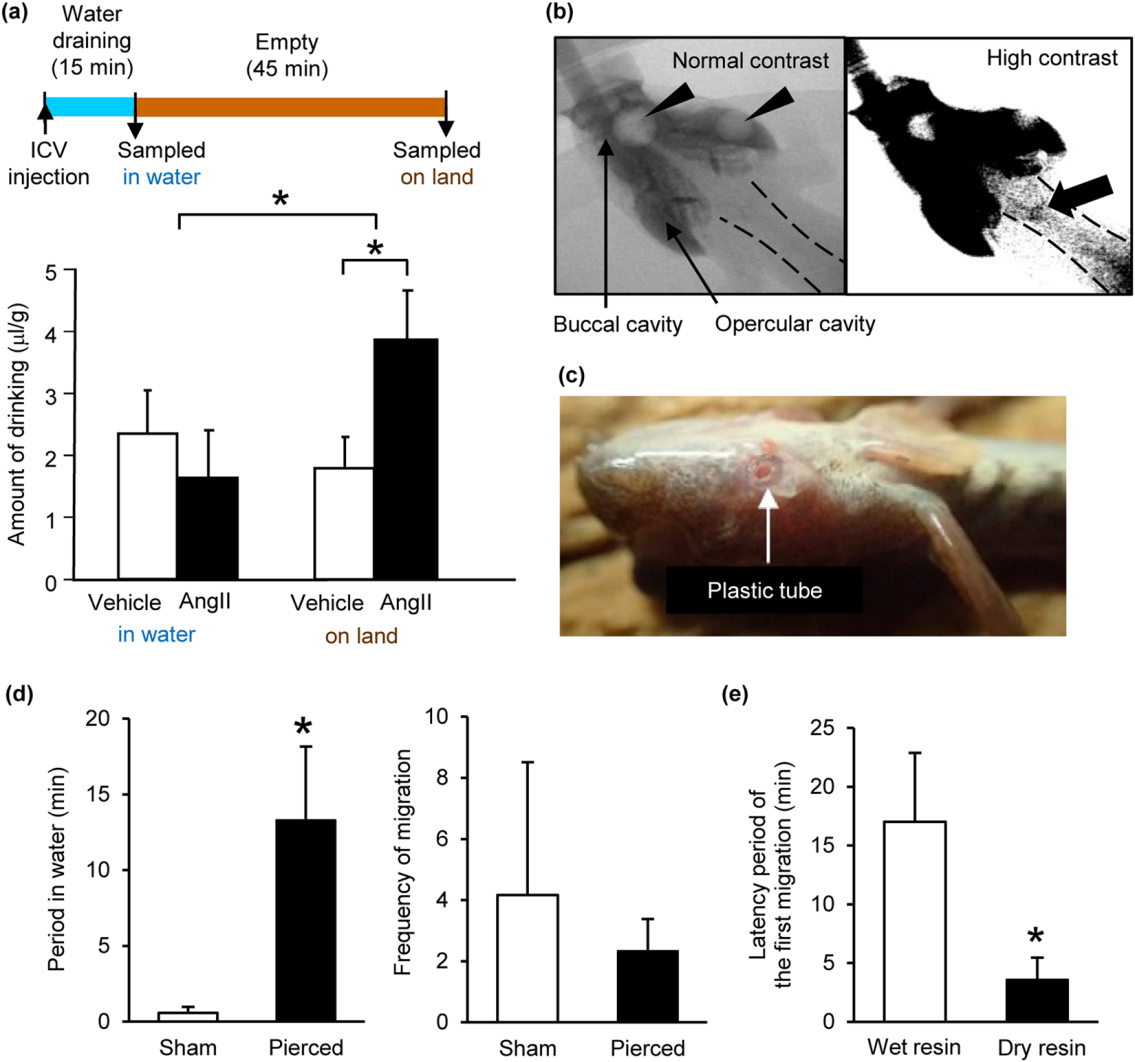
Loss of buccal/opercular water by angiotensin II (AngII) and its induction of migration to water. (**a**) The effect of AngII on amount of drinking on land. The diagram shows the experimental design. After the intracerebroventricular injection of AngII or vehicle, water was completely drained out for 15 min. Mudskippers could hold water in the buccal/opercular cavity and drink it even in the empty tank. Two-way factorial ANOVA was used for statistical analysis and the interaction was significant. *P < 0.05, *n* = 6. (**b**) X-ray images showing the presence of water (deep gray) in the buccal and opercular cavity. Arrow heads indicate air in these cavities. A thick arrow in the high-contrast image shows water in the anterior part of the oesophagus before swallowing (see Supplementary Movie 1). Broken lines show the gastrointestinal tracts. (**c**) A picture showing a tube which pierced the operculum to artificially eliminate buccal/opercular water. (**d**) Amphibious behaviour of ‘pierced’ mudskippers for 30 min. *P < 0.05 with unpaired Wilcoxon signed-rank test, *n* = 6. (**e**) Effect of decreased buccal/opercular water by placing water-absorptive dry resin (‘dry resin’) on amphibious behaviour. Controls were treated with the water pre-absorbed resin (‘wet resin’). *P < 0.05 with paired Wilcoxon signed-rank test, *n* = 8. Data are shown as mean ± s.e.m.

We examined whether the swallowing of buccal/opercular water on the land area is enhanced by AngII and induces migration into water. On land, ICV administration of AngII induced swallowing of more water stored in the buccal/opercular cavity than controls (Fig. 4a). X-ray observation using a contrast medium visualized how buccal/opercular water was swallowed (Supplementary Movie 1). When the sphincter at the anterior oesophagus was relaxed, water held in the buccal/opercular cavity and in the most anterior part of the oesophagus (Fig. 4b) flowed into the intestine; the mudskipper does not have a stomach and the oesophagus is connected directly to the proximal intestinal swelling. When the mudskipper had little water in the buccal/opercular cavity, the immediate migration to water and refilling were observed (Supplementary Movie 2).

We further examined the effect of artificial removal of buccal/opercular water on the migratory behaviour. The removal of buccal/opercular water by piercing holes in both sides of the opercular skin (Fig. 4c) increased the period in water compared with controls; the frequency of migration between water and land areas did not change (Fig. 4d). In addition, the removal of buccal/opercular water by placing water-absorptive dry resin in the buccal/opercular cavity decreased the latency of the first migration into water compared with controls (Fig. 4e).

## Discussion

The present study first demonstrated that AngII induced thirst to motivate mudskipper fish to move to water for drinking as shown in many tetrapods^27^. Histological analyses indicated that AngII acts primarily on the hindbrain CVO (the AP) rather than the forebrain CVO (the PPa). On the grounds that mudskippers store water in the buccal/opercular cavity on land^23,24^, we hypothesized and demonstrated herein that AngII induces the swallowing of buccal/opercular water by acting on the AP and the loss of buccal/opercular water motivates them to migrate into water for refilling and drinking. In tetrapods, systemic AngII directly acts on the forebrain to induce thirst^10,11,28^, and such thirst is categorized as ‘primary drinking’^1^ or ‘general thirst’^29^. Another type of thirst evoked by oropharyngeal cues (e.g., dry mouth) is considered to be ‘secondary drinking’ or ‘local thirst’^1,29^, and has only been revealed recently as ‘anticipatory thirst’ which occurs when water balance is normal but future deficits are predicted^30,31^. In the mudskipper, the migration to water was more directly stimulated by the sensory detection of buccal/opercular drying, and thus categorized as ‘local thirst’. Taken together, we compared the thirst mechanism of the mudskipper with the known mechanisms of terrestrial tetrapods and attempted to offer a view of thirst evolution during terrestrialization of vertebrates.

ICV injection of AngII induced migration to water in the mudskipper. The ICV procedure was chosen because this route is more consistent than peripheral injection for inducing this behaviour. Also in eels, ICV injection of AngII increases drinking rate more potently than does peripheral injection^32^. The dipsogenic effect of AngII injected into the circulation is only transient, and it is likely that the dipsogenic effect is masked by the short half-life of AngII in blood (ca. 6 min)^33^ as well as a secondary inhibition induced by the baroreflex^15^. In the present study, the effect of ICV AngII on the migration to water was not dose-dependent and 3 × 10^−8^ M was the most effective for 15–60 min after the injection, a duration which is similar to that shown in eels^32^. We speculate that the significant responses at 4 hours or 8 hours after the injection may be a secondary effect due to increased secretion of other hormones such as vasotocin and cortisol^34,35^ since these hormones also prolong the period spent in water^36,37^.

**Figure 5:**
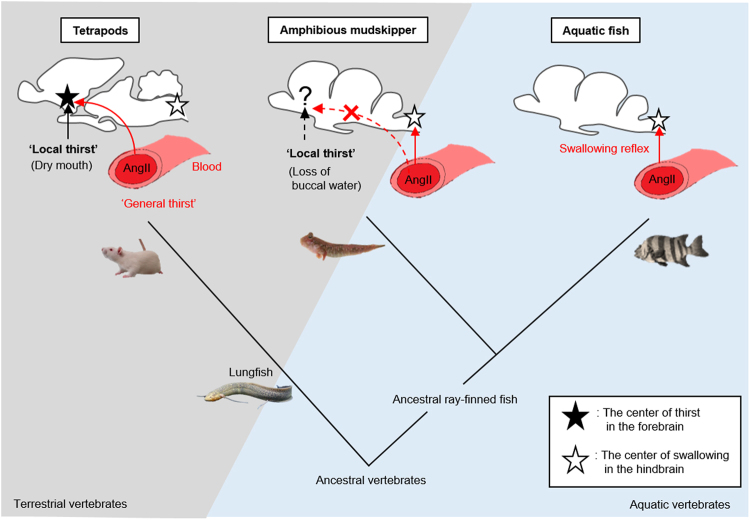
Schematic diagram showing evolution of the brain mechanisms of thirst. Black and white stars indicate the brain regions controlling thirst and swallowing reflex, respectively. In terrestrial tetrapods, systemic angiotensin II (AngII) directly acts on the forebrain (i.e., the center of emotional behaviour) to induce thirst (‘general thirst’), and oropharyngeal signals (e.g., dry mouth) also induce thirst (‘local thirst’). These signals are integrated in the forebrain and regulate motivation to move to water. In aquatic and the amphibious ray-finned fish, AngII acts primarily on the hindbrain (i.e., the reflex center) to induce swallowing of buccal water. In addition, in the amphibious mudskipper, loss of buccal water on land induces ‘local thirst’ to evoke migration to water, possibly through the forebrain, although the neural basis is not identified. Because ‘local thirst’ exists both in tetrapods and in the amphibious fish, this thirst appears to be important for invasion onto the land during the evolution of vertebrates. It is assumed that agnathan, cartilaginous fish, and possibly ancestral vertebrates whose body fluids are isosmotic have not developed the regulation of drinking.

The phylogenetically distant vertebrates (ray-finned fish and tetrapods) appeared to acquire ‘local thirst’ that expedited a series of drinking behaviours and limited the time when they were exposed to a desiccative environment (Fig. 5). Aquatic fish always have water available for drinking through the mouth. They only needed a system in which AngII induced swallowing directly at the hindbrain level rather than the thirst system mediated by the forebrain^15^. The mudskipper, which evolved from aquatic ray-finned fish to invade the terrestrial environment^18,38^, may have acquired the ability to detect the buccal/opercular drying (i.e., ‘local thirst’), in addition to the AngII-induced swallowing at the hindbrain (Fig. 5). The mudskipper is unique among terrestrial animals to store water in the buccal/opercular cavity and in the anterior part of the oesophagus on land (see Supplementary Movie 1) to maintain the function of gills as the respiratory and osmoregulatory organ^23^. Without the migration to water, the mudskipper can swallow water as long as buccal/opercular water is held. When buccal/opercular water is lost by swallowing, the refilling behaviour is induced to moisten the gills and the buccal cavity, possibly for the maintenance of the structure and function of the gills^24^. Together with no histochemical evidence for the forebrain site of AngII action, mudskippers do not appear to have acquired ‘general thirst’. Local sensation plays an important role in mudskipper drinking behaviour, which has often been missed in studies of drinking in vertebrates because the dipsogenic/antidipsogenic effects of hormones are usually more potent than local sensation^1^. In contrast, terrestrial tetrapods with pulmonary respiration cannot physically store water readily available for drinking, and must move to water via the motivation signal based on the forebrain by systemic AngII for drinking^5,15,39^. Thus, ‘general thirst’ is a characteristic of terrestrial tetrapods and may have been acquired when they lost their gills during the evolution (Fig. 5).

Oropharyngeal water and its drying are reported to be detected by afferent fibers of the Vth, IXth and Xth cranial nerves in mammals^40,41^ and the signals are relayed to the forebrain, such as the SFO and the limbic system, to regulate thirst^3,30,40^. Also in eels, the Xth cranial nerve (vagus nerve) that innervates the buccal/opercular cavity is critical for drinking in response to Cl^−^ in the buccal/opercular water^42^. Since our X-ray movies showed that the mudskipper searched for water immediately when water was absent within the buccal/opercular cavity, the cranial nerves may also sense the presence of water. In mammals, several studies have demonstrated that such neural mechanisms are involved in the regulation of antidiuretic vasopressin secretion as well as the thirst regulation without changes in systemic water balance^40,43–47^. The vasopressin neurons that innervate the OVLT have recently been shown to play an important role in ‘anticipatory thirst’^48^. In the mudskipper, ICV injection of vasotocin, the teleost homologue of vasopressin, strongly induced migration to water^37^ and drinking (Supplementary Fig. 3b), suggesting an involvement of vasotocin neurons in the regulation of ‘local thirst’ also in the mudskipper. Therefore, it is possible that the signal derived from the cranial nerves is relayed to the forebrain, similarly to the case in mammals, to induce ‘local thirst’ in the mudskipper. Since the SFO neurons monitor blood factors for the regulation of ‘general thirst’, as well as mediate the sensation of oropharyngeal cues in mammals^30^, the mammalian thirst mechanisms are highly complex. Although the neural basis of thirst is not identified in the mudskipper forebrain, the ‘local thirst’ regulation in the forebrain appears to be independent from the major site of AngII action in the hindbrain (Fig. 5). Therefore, the ‘local thirst’ regulation can be specifically analyzed in this fish by excluding the impact of ‘general thirst’. Together with the less complicated fish brain and recent publication of genome databases of mudskipper species^38^, the mudskipper might become a unique and excellent model to investigate this mechanism.

In the present study, we suggest that ‘local thirst’ in the mudskipper and tetrapods represents a first case of convergent evolution of thirst mechanisms and may play a critical role in the early stage of terrestrialization of vertebrates. The mudskipper lives in brackish tidal flats, where many organisms emerged onto land and diversified into the terrestrial habitats^17,18,49,50^. Tide pools often dry up, as suggested to have happened in the seasonal drought of the Devonian period, and such environments have been suggested to impose selection pressures for ‘walking’ and the acquisition of limbs to move to adjacent pools to acquire water^51^. Similarly, such selection pressures may have led to the evolution of a neural basis of ‘local thirst’. Understanding the early emergence of ‘local thirst’ should provide a fresh insight into the characters acquired during the terrestrialization of vertebrates. The Devonian vertebrates such as *Acanthostega* and *Tiktaalik* are possible ancestral tetrapods and still possessed gills^52,53^. The buccal/opercular water held by the mudskipper and possibly by ancestral tetrapods, may have originally prevented the gills from desiccation, but also allowed them to stay on land longer as a source of drinking water. It conferred protection against dehydration and thus, might be a preadaptation for terrestrialization in vertebrates. As discussed above, it was suggested in mammals that ‘local thirst’ functions as ‘anticipatory thirst’ to prevent future dehydration^30^. However, more evidence is required in various vertebrates including aquatic ray-finned fish and amphibious lungfish to generalize the function and mechanisms of ‘local thirst’ in the evolution (Fig. 5).

## Methods

### Animals

One year-old mudskippers of both sexes (*P. modestus*) weighing 3 to 5 g were collected from the estuary of the Fujii River, which flows into the Inland Sea of Seto (34°N: 134°E). No sex differences have been found in their amphibious behaviour in our previous reports^36,37,54^. Fish were acclimated for 2–5 weeks in laboratory tanks (3 L). Since these fish were collected from brackish water, tank water was diluted seawater (10 ppt, 149 mM Na^+^, 176 mM Cl^−^, 3.8 mM Ca^2+^, 346 mOsml/kg), which is almost isotonic to mudskipper plasma. All specimens were maintained at room temperature of 22–25 °C under a daily photoperiod cycle of 12-h light/12-h dark (lights on at 7:00 a.m.) and were fed daily with Tetrafin flakes (TetraWerke, Melle, Germany). Small plates were placed in each tank to allow mudskippers an opportunity to climb onto them. Fish were anesthetized in 0.01% tricaine methanesulfonate (Sigma, Tokyo, Japan) neutralized with sodium bicarbonate before handling. All experiments were approved by the Animal Experiment Committee of the University of Tokyo, Okayama University and National Cerebral and Cardiovascular Center, and performed in accordance with the manuals prepared by the committees.

### Cloning of angiotensinogen and AT1 cDNAs

The procedure for cDNA cloning was modified from the previous study^55^. Single-stranded cDNAs were prepared from RNA of the liver and the brain for angiotensinogen and AngII receptor type 1 (*at1*), respectively, using high capacity cDNA Reverse Transcription kit (Thermo Fisher Scientific, MA, USA). The 5′ and 3′ regions of the cDNAs were amplified using SMARTer^TM^ RACE cDNA Amplification Kit (Clontech, Takara Bio USA, CA, USA). A degenerate primer (GCYGCYGAGAAYGTCAGCTGYGA) and a gene specific primer (AAGTGACAGATATTCCGGGAATTG) were used for 3′ RACE and 5′ RACE of angiotensinogen, respectively. For *at1* cloning the AT1, a partial sequence was first cloned by a nested PCR with four degenerate primers (TBRTSGGDAAYASCATGGTGGT, GACCGBTACCTKGCYATYGTSCA, GCAGCTRTTRAMGTARGCDATGCA, TGSACRATRGCMAGGTAVCGGTC). Then gene specific primers (GAAGAGCTCCCAGTCCCGGGACG and CAGTCATCGTGGCCCACATGGGC) were used for 3′ and 5′ RACE, respectively. Finally, cDNAs encompassing the whole coding regions were amplified using gene-specific primers designed based on the partial sequences determined by the RACE method. Amplified products were sequenced by an ABI3130*xl* DNA sequencer (Applied Biosystem, Foster City, CA, USA). Deduced amino acid sequences of angiotensinogen and AT1 were aligned with those of other vertebrates and phylogenetic trees were generated by the neighbor-joining method using the ClustalW program (version 2.1) in DDBJ at http://clustalw.ddbj.nig.ac.jp/. For comparison of amino acid sequences of mudskipper AT1 with those of other members of the same family, AT1, angiotensin receptors type 2 (AT2), bradykinin receptors, and apelin receptors were included in the phylogenetic tree.

### ICV injection of AngII

Mudskipper AngII (NRVYVHPF) synthesized by Peptide Institute (Osaka, Japan) was used in this study. ICV injection has been routinely performed in the mudskipper^36,37,54^. Anesthetized fish were injected post-orbitally along the midline into the third ventricle with 0.1 μl/g volume. AngII concentrations were from 3 × 10^−12^ to 3 × 10^−6^ M, according to the preliminary studies and published reports^36,37,56^. Injection of artificial cerebrospinal fluid (vehicle) served as controls. Evans blue (0.1%) was used to confirm the success of ICV injection. To minimize the leakage from the injection site, 30 sec was allowed to elapse after each injection. Fish were fully recovered from anesthetization in 1–2 min.

### Testing for amphibious behaviour

Immediately after injection of AngII, each fish was placed in the water area of experimental tank (Fig. 2a) as reported previously^36,37^. The land area was made of plastic mesh, and care was taken to ensure that there was minimum water on the area. Water in the tank was constantly aerated. The period in water and the frequency of migration between water and land area (defined as the ‘frequency of migration’) were recorded for 8 h.

### *In situ* hybridization of *at1*

The procedure was previously described by Hasegawa *et al*.^57^ and details are described in the Supplementary information.

### c-Fos immunohistochemistry

The procedure was previously described by Hamasaki *et al*.^25^ and details are described in the Supplementary information.

### Swallowing of buccal/opercular water induced by AngII on land

After ICV injection of 3 × 10^−8^ M AngII or vehicle, fish were transferred to a 1,000-ml Büchner funnel (TPP rapid 1000, TPP Techno Plastic Products, Switzerland) containing 0.004% phenol red (Sigma-Aldrich) in aerated diluted seawater (500 ml). The water was completely eliminated by aspiration without disturbing the fish for 15 min after the injection, and the amount of ingested water was measured (‘in water’, *n* = 6 each). In another group, mudskippers were subsequently allowed to stay without water for an additional 45 min to test whether they drink water held in the buccal/opercular cavity (‘on land’, *n* = 6 each).

Fish were sacrificed after deep anesthesia, and the amount of water in the whole gastrointestinal tract was measured according to the method of Kobayashi *et al*.^58^. Briefly, the whole tracts were removed on a petri dish and washed by 1 ml saline. The 0.5-ml samples were mixed with 0.5 ml 5% trichloroacetic acid (Sigma-Aldrich), and centrifuged at 10,000 rpm for 5 min in a centrifuge (Sakuma M-160-IV, Tokyo, Japan). The supernatant was mixed with 0.5 ml of 1 M NaOH, and absorbance was determined at 550 nm wave length by a spectrophotometer (DU640, Beckman Coulter, CA, USA).

### Artificial removal of buccal/opercular water

First, a hole was drilled in each side of opercula after anesthesia, and a polyethylene tube (0.8 mm ID, 1.6 mm OD, Natsume, Tokyo, Japan) was inserted into the hole (Fig. 4c). The site of the hole was determined according to the previous report that showed the water storage capacity of the opercular cavity^59^. Sham controls were prepared by sealing the tube with instant glue. After surgery, fish were allowed to recover overnight. Amphibious behaviour was tested in the experimental tank (Fig. 2a) as described above.

Alternatively, buccal/opercular water was removed by filling the cavity with 0.05 g of dry Sephadex G-25 resin (GE health care, Tokyo, Japan) after light anesthesia. As controls, fish were treated with the same amount of the 0.9%-NaCl pre-absorbed resin. After treatment, fish were placed on the land area to test amphibious behaviour (Fig. 2a). Following the recovery from anesthesia, the latency for migration into water was recorded. In this experiment, the same fish were used for both groups (*n* = 8).

### X-ray observation of drinking behaviour

The real-time drinking behaviour was observed with a microfocus X-ray angiography system (HITEX MFX-80, Hitex, Osaka, Japan) by modifying the methods used in our research^60^. In the first trial, mudskippers were put into 100-mm polystyrene culture dishes with covers (Fig. 4b and Supplementary Movie 1). In the dish, the mudskipper could move horizontally, but could not jump. From the side of the dish, diluted seawater containing iodinated contrast medium (Iomeron 350; Bracco-Eisai, Tokyo, Japan) to make the water radiopaque was gradually supplied through a tube using a syringe pump (PHP4400 Programmable, Harvard Apparatus, MA) to deliver fluid into the X-ray angiography system. In the next trial, the mudskipper was placed on top of a 96-well microplate (Corning, NY), where one hole contained 300 μl of contrast medium and other holes were covered by transparent tape (Supplementary Movie 2). During each cine-scan, monochromatic X-rays at 80 kV passed through the mudskipper, and the images were recorded at 30 frames/s for a few minutes. Still images taken by the X-ray camera were converted to movies using Image J.

### Statistics analyses

The data are expressed as means ± s.e.m. Kyplot 5.0 (KyensLab, Tokyo, Japan) was used for statistics analyses. Time course data were analyzed by two-way ANOVA followed by an appropriate post-hoc test. Other data were analyzed by Student-*t* test or non-parametric Wilcoxon single-rank test. The amount of water intake was analyzed with two-way ANOVA, followed by *t*-test. Effects of AngII or vasotocin on drinking rate were analyzed with one-way ANOVA followed by Dunnett’s post-hoc test wherever appropriate. All data were checked for normal distribution and equal variance.

### Data Availability

The data that support the findings of this study are available from the corresponding author upon request.

## Electronic supplementary material

Supplementary Information

Supplementary Movie 1

Supplementary Movie 2

## References

[1] Fitzsimons, J. T. *The physiology of thirst and sodium appetite* (Cambridge Univ. Press Camridge, 1979). 400173

[2] Robertson, G. L. Disorders of thirst in man. In *Thirst* (eds Ramsay, D. J. & Booth, D. A.) 453–477 (Springer London, 1991).

[3] Denton D (1999). Neuroimaging of genesis and satiation of thirst and an interoceptor-driven theory of origins of primary consciousness. Proc. Natl. Acad. Sci. USA.

[4] Fitzsimons JT (1998). Angiotensin, thirst, and sodium appetite. Physiol. Rev..

[5] Kobayashi H, Uemura H, Wada M, Takei Y (1979). Ecological adaptation of angiotensin-induced thirst mechanism in tetrapods. Gen. Comp. Endocrinol..

[6] McKinley MJ, Johnson AK (2004). The physiological regulation of thirst and fluid intake. News. Physiol. Sci..

[7] Takei Y (2000). Comparative physiology of body fluid regulation in vertebrates with special reference to thirst regulation. Jpn. J. Physiol..

[8] Johnson AK, Buggy J (1978). Periventricular preoptic-hypothalamus is vital for thirst and normal water economy. Am. J. Physiol..

[9] McKinley, M. J. *et al*. *The sensory circumventricular organs of the mammalian brain: subfornical organ, OVLT and area postrema* (Adv. Anat. Embryol. Cell Biol. 172, 2003). 10.1007/978-3-642-55532-9 12901335

[10] Simpson JB, Routtenberg A (1973). Subfornical organ: site of drinking elicitation by angiotensin II. Science.

[11] Takei Y (1977). The role of the subfornical organ in drinking induced by angiotensin in the Japanese quail. Coturnix coturnix japonica. Cell Tissue Res..

[12] Hoff KvS, Hillyard SD (1991). Angiotensin II stimulates cutaneous drinking in the toad *Bufo punctatus*. Physiol. Zool..

[13] Takei Y, Hirano T, Kobayashi H (1979). Angiotensin and water intake in the Japanese eel. Anguilla japonica. Gen. Comp. Endocrinol..

[14] Nobata S, Takei Y (2011). The area postrema in hindbrain is a central player for regulation of drinking behavior in Japanese eels. Am. J. Physiol. Regul. Integr. Comp. Physiol..

[15] Nobata S, Ando M, Takei Y (2013). Hormonal control of drinking behavior in teleost fishes; insights from studies using eels. Gen. Comp. Endocrinol..

[16] Takei Y (2015). From aquatic to terrestrial life: evolution of the mechanisms for water acquisition. Zoolog. Sci..

[17] Graham JB, Lee HJ (2004). Breathing air in air: in what ways might extant amphibious fish biology relate to prevailing concepts about early tetrapods, the evolution of vertebrate air breathing, and the vertebrate land transition?. Physiol. Biochem. Zool..

[18] Ord TJ, Cooke GM (2016). Repeated evolution of amphibious behavior in fish and its implications for the colonisation of novel environments. Evolution.

[19] Clayton DA (1993). Mudskippers. Oceanogr. Mar. Biol. Ann. Rev..

[20] Graham, J. B. *Air-breathing fishes: evolution, diversity, and adaptation* (Academic Press San Diego, 1997).

[21] Ishimatsu, A. & Gonzales, T. T. Mudskippers: front runners in the modern invasion of land. In *The Biology of* Gobies (eds Patzer, R. A., Van Tassell, J. K., Kovacic, M., Kappor B. G.) 609–638 (Science, 2011).

[22] Sakamoto T, Yasunaga H, Yokota S, Ando M (2002). Differential display of skin mRNAs regulated under varying environmental conditions in a mudskipper. J. Comp. Physiol. B.

[23] Sayer MDJ (2005). Adaptations of amphibious fish for surviving life out of water. Fish Fish.

[24] Tamura SO, Morii H, Yuzuriha M (1976). Respiration of the amphibious fishes *Periophthalmus cantonensis* and *Boleophthalmus chinensis* in water and on land. J. Exp. Biol..

[25] Hamasaki S, Mukuda T, Kaidoh T, Yoshida M, Uematsu K (2016). Impact of dehydration on the forebrain preoptic recess walls in the mudskipper, *Periophthalmus modestus*: a possible locus for the center of thirst. J. Comp. Physiol. B.

[26] Wong, M. K. & Takei, Y. Molecular and evolutionary perspectives of the renin-angiotensin system from lamprey. *Gen. Comp. Endocrinol*., in Press, 10.1016/j.ygcen.2017.01.031 (2017). 10.1016/j.ygcen.2017.01.031 28161438

[27] Kobayashi, H. & Takei, Y. *The renin-angiotensin system: comparative aspects* (Springer Science Berlin, 1996).

[28] Uchiyama, M. Angiotensin II and Water Balance in Amphibians. in *Sodium and* Water Homeostasis (ed. Hyndman K. A. & Pannabecker T. L.) 73–90 (Springer, 2015).

[29] Cannon WB (1918). Croonian Lecture: the physiological basis of thirst. Proc. R. Soc. Lond. B Biol. Sci..

[30] Zimmerman CA (2016). Thirst neurons anticipate the homeostatic consequences of eating and drinking. Nature.

[31] Krashes MJ (2016). Forecast for water balance. Nature.

[32] Kozaka T, Fujii Y, Ando M (2003). Central effects of various ligands on drinking behavior in eels acclimated to seawater. J. Exp. Biol..

[33] Wong MK, Takei Y (2012). Changes in plasma angiotensin subtypes in Japanese eel acclimated to various salinities from deionized water to double-strength seawater. Gen. Comp. Endocrinol..

[34] Saavedra JM (1992). Brain and pituitary angiotensin. Endocr. Rev..

[35] Arnold-Reed DE, Balment RJ (1994). Peptide hormones influence *in vitro* interrenal secretion of cortisol in the trout. Oncorhynchus mykiss. Gen. Comp. Endocrinol..

[36] Sakamoto T (2011). Corticosteroids stimulate the amphibious behavior in mudskipper: Potential role of mineralocorticoid receptors in teleost fish. Physiol. Behav..

[37] Sakamoto T (2015). Neurohypophysial hormones regulate amphibious behaviour in the mudskipper goby. PLoS One.

[38] You X (2014). Mudskipper genomes provide insights into the terrestrial adaptation of amphibious fishes. Nat. commun..

[39] Hillyard SD, Hoff KvS, Propper C (1998). The water absorption response: a behavioral assay for physiological processes in terrestrial amphibians. Physiol. Biochem. Zool..

[40] Norgren, R. Sensory detection of water. In *Thirst* (ed. Ramsay, D. J. & Booth, D. A.) 221–231 (Springer London, 1991).

[41] Norgren R, Smith GP (1988). Central distribution of subdiaphragmatic vagal branches in the rat. J. Comp. Neurol..

[42] Mayer-Gostan N, Hirano T (1976). The effects of transecting the IXth and Xth cranial nerves on hydromineral balance in the eel *Anguilla anguilla*. J. Exp. Biol..

[43] Figaro MK, Mack GW (1997). Regulation of fluid intake in dehydrated humans: role of oropharyngeal stimulation. Am. J. Physiol..

[44] McKenna K, Thompson C (1998). Osmoregulation in clinical disorders of thirst appreciation. Clin. Endocrinol. (Oxf.).

[45] Stricker EM, Hoffmann ML (2005). Inhibition of vasopressin secretion when dehydrated rats drink water. Am. J. Physiol. Regul. Integr. Comp. Physiol..

[46] Stricker EM, Stricker ML (2011). Pre-systemic controls of fluid intake and vasopressin secretion. Physiol. Behav..

[47] Thrasher TN, Nistal-Herrera JF, Keil LC, Ramsay DJ (1981). Satiety and inhibition of vasopressin secretion after drinking in dehydrated dogs. Am. J. Physiol..

[48] Gizowski C, Zaelzer C, Bourque CW (2016). Clock-driven vasopressin neurotransmission mediates anticipatory thirst prior to sleep. Nature.

[49] Long JA, Gordon MS (2004). The greatest step in vertebrate history: a paleobiological review of the fish-tetrapod transition. Physiol. Biochem. Zool..

[50] Schultze, H. P. The fossil record of the intertidal zone. In *Intertidal Fishes: Life in Two Worlds* (eds Horn, M. H., Martin, K. L. M. & Chotkowski, M. A.) 373–392 (Academic Press San Diego, 1998).

[51] Romer AS (1967). Major steps in vertebrate evolution. Science.

[52] Clack, J. A. *Gaining ground: the origin and evolution of tetrapods* (Indiana Univ. Press, 2002).

[53] Daeschler EB, Shubin NH, Jenkins FA (2006). A Devonian tetrapod-like fish and the evolution of the tetrapod body plan. Nature.

[54] Kagawa N (2013). Potential roles of arginine-vasotocin in the regulation of aggressive behavior in the mudskipper (*Periophthalmus modestus*). Gen. Comp. Endocrinol..

[55] Nobata S, Ventura A, Kaiya H, Takei Y (2010). Diversified cardiovascular actions of six homologous natriuretic peptides (ANP, BNP, VNP, CNP1, CNP3, and CNP4) in conscious eels. Am. J. Physiol. Regul. Integr. Comp. Physiol..

[56] Ogoshi M, Nobata S, Takei Y (2008). Potent osmoregulatory actions of homologous adrenomedullins administered peripherally and centrally in eels. Am. J. Physiol. Regul. Integr. Comp. Physiol..

[57] Hasegawa K (2016). Sulfate transporters involved in sulfate secretion in the kidney are localized in the renal proximal tubule II of the elephant fish (*Callorhinchus milii*). Am. J. Physiol. Regul. Integr. Comp. Physiol..

[58] Kobayashi H (1983). Drinking induced by angiotensin II in fishes. Gen. Comp. Endocrinol..

[59] Michel KB, Heiss E, Aerts P, Van Wassenbergh S (2015). A fish that uses its hydrodynamic tongue to feed on land. Proc. R. Soc. Lond. B: Biol. Sci..

[60] Sonobe T, Tsuchimochi H, Schwenke DO, Pearson JT, Shirai M (2015). Treadmill running improves hindlimb arteriolar endothelial function in type 1 diabetic mice as visualized by X-ray microangiography. Cardiovasc. Diabetol..

