# Supplementary material for: Drinking by amphibious fish: convergent evolution of thirst mechanisms during vertebrate terrestrialization
Source: Sci Rep. 2018 Jan 12;8:625. doi: 10.1038/s41598-017-18611-4 (PMC5766589; doi:10.1038/s41598-017-18611-4)
Supplement: Supplementary file 1 — Supplementary Information [file 41598_2017_18611_MOESM1_ESM.pdf]

Supplementary Information

Title: Drinking by amphibious fish: convergent evolution of thirst mechanisms during vertebrate terrestrialization

Author affiliation: Yukitoshi Katayama[1,*], Tatsuya Sakamoto[2], Kazuhiro Saito[2], Hirotsugu Tsuchimochi[3], Hiroyuki Kaiya[4], Taro Watanabe[1], James T. Pearson[3], Yoshio Takei[1]

[1]Laboratory of Physiology, Atmosphere and Ocean Research Institute, University of Tokyo, 5-1-5 Kashiwanoha, Kashiwa, Chiba 277-8564, Japan

[2]Ushimado Marine Institute, Faculty of Science, Okayama University, 130-17 Kashino, Setouchi, Okayama 701-4303, Japan

[3]Department of Cardiac Physiology, National Cerebral and Cardiovascular Center Research Institute, 5-7-1 Fujishirodai, Suita, Osaka 565-8565, Japan

[4]Department of Biochemistry, National Cerebral and Cardiovascular Center Research Institute, 5-7-1 Fujishirodai, Suita, Osaka 565-8565, Japan

[*]**Corresponding author:** Yukitoshi Katayama, Laboratory of Physiology, Atmosphere and Ocean Research Institute, University of Tokyo, 5-1-5 Kashiwanoha, Kashiwa, Chiba 277-8564, Japan

E-mail address: katayama3g@aori.u-tokyo.ac.jp

Tel: +81-4-7136-6204, Fax: +81-4-7136-6206

Supplementary Methods

***In situ* hybridization of AT1.** The brains were fixed in 4% paraformaldehyde in phosphate buffer (PB) and embedded in Palaplast (McCormick Scientific, Richmond, IL, USA). Serial sections were cut at 7 μm to obtain cross sections and mounted onto MAS-coated slides (Matsunami Glass, Osaka, Japan). The cDNA fragment (1095 bp) specific to *at1* was amplified using gene-specific primers (ATGAAGAACATAACCTCAGGAGCAG, TCATTTAATGTCAGAGGAAGACTTG) and used to synthesize digoxigenin-labeled antisense and sense cRNA probes (DIG RNA Labeling Kit; Roche Applied Science, Mannheim, Germany) according to the manufacturer's protocols. The rehydrated, rinsed sections were treated with 5 mg/ml proteinase K (Sigma-Aldorich, MO, USA) in Tris-EDTA buffer [100 mM tris (hydroxymethyl) aminomethane and 50 mM ethylenediaminetetraacetic acid, pH 7.5] and then hybridized with 1 μg/ml DIG-labeled cRNA probes in hybridization buffer [50% formamide, 5 × Saline Sodium Citrate Buffer (5 × SSC, 750 mM NaCl and 75 mM sodium citrate, pH 7.0), 40 μg/ml bovine calf thymus DNA] at 58°C for 40 h. After hybridization, sections were washed in 2 × SSC for 30 min at room temperature, followed by 1 × SSC for 1 h at 65°C, and finally in 0.1 × SSC for 1 h at 65°C. Following immunohistochemical reaction with alkaline phosphatase-conjugated anti-DIG antibody (Roche Applied Science, Upper Bavaria, Germany), hybridization signals were visualized with 4-nitro blue tetrazolium chloride (450 mg/ml) and X-phosphate/5-bromo-4-chloro-3-indolyl-phosphate (175 mg/ml) after 1 week at room temperature. Stained sections

were dehydrated with ethanol, cleared with xylene, coverslipped, and observed under a microscope with image sensor (FSX100, Olympus, Tokyo, Japan).

c-Fos immunohistochemistry. The brains were dissected out 1 h after ICV injection of AngII or vehicle and fixed in 4% paraformaldehyde in PB. The fixed brains were cut at 7 μm. The sections were immersed in methanol containing 0.3% H_2O_2 for 30 min at room temperature. After rinsing in phosphate buffered saline (PBS), the sections were incubated with proteinase K (10 mg/ml in PBS) for 10 min at room temperature. The sections rinsed thoroughly in PBS were pretreated with a blocking solution (2% normal goat serum, 0.01% NaN_3 in PBS) for 1 h at room temperature and incubated with a polyclonal antibody raised against human c-Fos (1:200, sc-253, SantaCruz, Dallas, TX, USA) for 12 h at 4°C. Specificity of the antibody against c-Fos protein of the mudskipper was checked by Western blotting analysis [25]. After rinsing in PBS, the sections were treated with ABC Elite kit (PK-6101, Vector, Burlingame, CA, USA) according to the manufacturer's instruction. The sections rinsed in 0.1 M PB were immersed in 0.01% 3, 3' -diaminobenzidine tetrahydrochloride (DAB) in PB for 3 min to intensify colourization and then incubated in 0.01% DAB solution containing 0.01% H_2O_2 for 3 min in dark. Finally, the sections were rinsed in PB and distilled water, and immunoreactive c-Fos was examined. The photomicrographs were binarized by graphic software Image J (https://imagej.nih.gov/ij/) and the number of c-Fos positive neurons was counted manually and compared between the cohorts. The parvocellular preoptic area anterior part (PPa) was defined

from the rostral tip of the anteroventral region of the third ventricle up to the level where the corpus callosum emerged caudally. Every third sections was analyzed in order not to count positive cells twice.

Supplementary Figures

(a)

```
Mudskipper    NQHHKNLTIVKMKRFTLVLLLCSCIFCGSSANRVYVHPFNLFAAENV----------SCE 50
Medaka        MQRLQAPLLVVLL---------CC--CLSRANRVYVHPFYLFAAENV----------SCE 39
Zebrafish     MKMFLAFLFLSCF-------------AMARTNRVYVHPFNLFSSENI----------SCE 37
Elephant_fish MKWALCFLCLGM------------HVALIRSNRPHIHPFLLMY--------------VNK 34
Sea lamprey   MKLFILLLLAFC-----AALCSPCVGEEDYDDRPYMQPFHLIPPPL-----------SVQ 44
                                              *    ** *

Mudskipper    TLQTQTKSLE-TISM---------VPLDMN-------VLTPDIRNMSQV-DIQRQNIRETT 93
Medaka        TLHNQISKPLETLPV---------SPLDSE-------VLTPDNRDPSKV-NPQNITER--T 81
Zebrafish     VIQSEEHKPLETVHP---------LPPLPG-------STDPDPRTASAAESLKNLTQR--T 80
Elephant_fish TQSDDQVN---NKTFLPPLAEFLKLQARM-------VPD---SGSSDDEDQAHPKTKQIW 81
Sea lamprey   ATEQPLASNE-TWDYPEPLAPGQSPAASSEEGSSEEKGDERESHRGEGRRGRKDKYKSKT 103

Mudskipper    AVLAKLLNSVGLRM-YQALS-GKQQSS--------NTLLSPVNT--------YGSLVTFY 135
Medaka        AVLAGLLNPLGLRM-YVALS-RKQRSE--------NTLFSPVNT--------CGSLFSFY 123
Zebrafish     AVLAELQNSLGLRM-YQTLS-RTQKHT--------NTLLSPLNA--------FGALVTLY 122
Elephant_fish LKERN--TKLQAVAISQTENTVVLLNH--------AFPPNRCADLVLSPLQIYSSLAALS 131
Sea lamprey   QRIASAVNGLGFRLYKQVLGGAGPADNIFFSPLSIASALGVVAA-----------GANGS 152

Mudskipper    LGAAKKTARSYQLLLGLSSDT-DREDCVSLVDGHRVLRTLQSINSLVDDGPKDEI-STQV 193
Medaka        LGASKTTASYFLRLLGLSDTD-REE-CLSIVDGHKVLGTLQNIHSLVDDGPKEKI-TTQV 180
Zebrafish     LGASKKTAISYQQLLGLNLES-EQTDCAYFVDGHTVLRTLQAIS--AHVDESRKELRTLV 179
Elephant_fish LGAANATAQIFHDILGFTSSSQVTTANLREKDASVRQLN----DFLLSWLPGRYL-ST-R 185
Sea lamprey   TRAELDTALGFKELLHGKKKAKSMKYFARLNSALYRRSAGFELMGKNVVFSKKGL--WLY 210
                *   **      *

Mudskipper    WAFTPTHTQL-SVDFVHGTQDF-SDSSFIRSVDFSNTQETEQQVNSFVEKTSGGKVKNIF 251
Medaka        WAFARKGAQL-SRDFIQGTQDF-SDSSYIRGVDFSNPQETEKLLNSFVEKTSNGSLRNVF 238
Zebrafish     WTFVNSDADL-SKEFLRGTQDF-SDDSFVRSVDFSQAKDAEVEVNNFIQKTSDNKVKSMF 237
Elephant_fish NWIFVQRDIK-VLESFAVNAKEFYNVNLLAIDFTHTPLAEQLINRY-MHNASGGGRVKDP 243
Sea lamprey   RQFTRTVAHLFKSNVRSVDFGESKEAVELMNAYIEKVT-----SKKFTDVISDVDTATSL 265
                                                                 *

Mudskipper    T-ELNTSSNLXFLSSF--NFQGNWKTVFQAEKTSEQEFYVDETTTIMTPV--MMHTGP-- 304
Medaka        A-DLNSSSNILFLSSF--NFQGKWKTAFQPEKTSLQEFYVNPTTTVMSPM--MTRTGH-- 291
Zebrafish     K-GVTPKTDLLFASSV--HFKGNWKTAFQPEATSD--QDFWTQKNSSVQVPFMMHTGD-- 290
Elephant_fish VIGLSPTATMMSASYM--EFKGKWKKAF--ESHTTELQDFFIEEDRRIRVPTMSQAGWFQ 299
Sea lamprey   MIVNVIYFKGSWANKFEPDLTKNVRFW----VNSS---YSMMVPTMHQRAKLSYAQDR-- 316

Mudskipper    --YHYLNDKVRRCTVVKLSLSKRSYMLLVLPHEGTSLGEIE----SKLLTVISYWNENLQ 358
Medaka        --YHYLNDKLRRCTVLKLSLSKQAYMMLVLPHEGVNLSDIETEA-NLHTNVFSAWHQNLQ 348
Zebrafish     --YKYLDDAGRKCSIVRLGLSKRTFMLLVLPHEGASLQDIEKPLLTVIPTWLRHLKEKYL 348
Elephant_fish GIVAHEYTAIKLPMAGSTS-MTIVQPVKVDLMQKIHRKLPIRTIFEQLQSRFVKVSLP-- 356
Sea lamprey   ---KLRSTVIKLPYEGGASMLVIVPHRTEELPKVEESVSQEQLEEWLSLLGPSNHYVQL- 372

Mudskipper    EGLLELSLPKFSMSAVNNLHDLLANMDPEIEAKLLGAQAEFSQLSNTKPFTVYKALNKVT 418
Medaka        EGPLELSLPKLSTSSVNDLNELLTNMNPEIEAKLLGSEAEFSQLSNITLFSIDKAVNTVR 408
Zebrafish     ELSLPKFSLTAVTDLRSVLSE---MAVEKYLMG--SD-ASFRRMSSKENFTVDKVLNKVV 402
Elephant_fish ---------KFKLDTTYNIKDIFLRMKGP---DAFSSEADFSRLSNDGRLKPDKVTNNIN 404
Sea lamprey   ------SLPKFKISVSYDLKAYLSAMGMP---SMFSYGADLSRITGMQKLHVDKITHKSV 423
                                                    *              *

Mudskipper    FEMSEEGTDAQTKTQDG----GIPLKLSINRPFFFS-IIEEDSGAILMLGKIINPAL--- 470
Medaka        FDMSEEGAEPQDKKEAA----GVPLRLSFNRPFFFS-VIEGYNNAILLLGKITNPLL--- 460
Zebrafish     FEMTEGGSEVQNRTDDG----RAPHKVTFNRPFFFA-VVEGNSNAILMLGKIINPTA--- 454
Elephant_fish FELTE-DWEEPVAQGNVQF--NNATEIKINRPFLFH-VYDDVSNTLLFVGRVKNLTEYDQ 460
Sea lamprey   LHVNEEGTEAKAETVVGIMPISMPPTVTVDRPFVVLIYDEKTRAVIFMGRVA-DPKQ--- 479
                  *                         ***

Mudskipper    -
Medaka        -
Zebrafish     -
Elephant_fish N 461
Sea lamprey   -
```

(b)

```
Mudskipper_AT1     MKNITSGAE------SDGINLTCGMSGNHD------FIFTLXPIVYGCNX   38
Medaka_AT1         MQNQT-AWA------TKEINLTCGMSGSHK------FIFTLVPIVYSFNF   37
Zebrafish_AT1a     MENQT-SAM------SEDLHVNCSMSGRHG------FIFTFIPVVYGCNF   37
Japanese eel_AT1   MENLT-VGR------TEGIHITCNTSGRHS------YIYTLIPVVYGCNF   37
Zebrafish_AT1b     MDNVT-SDS------NTGLALACNMTGHHS------FIFTFIPVVYSFNF   37
River lamprey_AT1  MSGVD-VANESLAALHNGTAIKNLASERCPAAFTNPTTLTAVPVMYGLIF   49
                   *                                      *  *  *

Mudskipper_AT1     IIGLVGNSMVVAVIYCYMKLKTVAHIFVLNLAISDLTFLITLPMWATMTA   88
Medaka_AT1         IIGIIGNTMVVAVIYFYMKLKTVANIFVLNLAISDLTFLITLPMWATFTA   87
Zebrafish_AT1a     VIGIIGNSMVVAVIFRYMKLKTVANVFVLNLAISDLTFLITLPLWATFTA   87
Japanese eel_AT1   VIGIVGNSMVVAVIYCYMKLKTVANIFVLNLAVSDLTFLITLPMWATFTA   87
Zebrafish_AT1b     IIGLIGNSLVVAVIYFCLKLKTVANIFVFNLAVSDLTFLLTLPIWAISTA   87
River lamprey_AT1  VAGLVGNTMVVAVVLKYLKLRNVANVYIVNLATADLVFVTTLPLWAASVA   99
                     *  **  ****     **  **     ***  ** *  *** **   *

Mudskipper_AT1     TGYHWPFGGFLCKATAALASFNLYTSIFFLTALSIDRYLAIVHPVSSRRF   138
Medaka_AT1         TEYSWPFGGFLCKTSAGLVTFNLYTSVFFLTALSTDRYLAIVHPVQSRRF   137
Zebrafish_AT1a     TGYHWIFGAFLCKASAGMVIFNLYTSIFFLTALSIDRYLAIVHPVRSRRQ   137
Japanese eel_AT1   MGYNWPFGGFLCKASAGLTIFNLYTSIFFLTSLSIDRYLAIVHPVRSRQR   137
Zebrafish_AT1b     TGYHWLFGDLLCKTIAGMALLNLYTSIFLLTALSVDRYLAIVHPVQSRRC   137
River lamprey_AT1  RGYDWPFGGFLCRVSATVVSVNMYASILLLACMSVDRYLAIVRPLQSRGR   149
                     * * **  **   *     * * *   *   * ******* *  **

Mudskipper_AT1     RTVLYARITCVLIWLFALLLSVPIALTRDIHNINNRNLTVCAILHPTKDN   188
Medaka_AT1         RTVVYARITCVVIWLFAFVLSVPTALTRDVHDIKNPQTTVCGILHPKAEN   187
Zebrafish_AT1a     RTLFYANLTCVLIWLFALLLSAPTALSRDVYDIGNLTLCAV-----WHSS   182
Japanese eel_AT1   RTVVYARITCVLIWAFAFLLSLPTALSRDVFTINHPNTTVC-----GTLD   182
Zebrafish_AT1b     RTVIYARVTCVLVWVVSFGLSLPTAIIRGTHFIQDNNVTVC-----AIHH   182
River lamprey_AT1  RTRRRARAACVVVWVTAVLLSVPVA--VFRVTFVHGGRTVC------ALR   191
                   **   *   **  *     ** * *

Mudskipper_AT1     VQSLEQVLLAISLMKSLLGFLLPFIIIITCYCLIGRALLGARHIQKSSQS   238
Medaka_AT1         SERLKELLLAIGVLKSLLGFLVPFIIIITCYCLIGRALLRVKHIQKNSRS   237
Zebrafish_AT1a     KQI--HFLVTLSVLKSVLGFIVPFLIIFTCYCLIGRALLGSRGLLRKSVR   230
Japanese eel_AT1   KNELSHVLVAIGLMKSVLGFLIPFVIIVTCYCLIGRALLEARRVQSSRSR   232
Zebrafish_AT1b     KEDIRNVLAALSLMKSVFGFLLPITVILTCYCLIGRALPKARDIQRNARS   232
River lamprey_AT1  YPSARTWFLAMNVTRNVAGFLVPFLVIVTCYSLIGRTLLRRGGDVVRRDK   241
                                     **  *   * *** **** *

Mudskipper_AT1     RD-DEVLRMLAAAVLAXFVCWVPHQIFHFMQLLTQLS-KTDNCRLLEIID   286
Medaka_AT1         RD-DEVLHMLAAAVLAFFLCWAPHQVFHFMQLLTQQILVENCTMLEII-D   285
Zebrafish_AT1a     SREDETLRMIAATVLAFFVCWAPHQAFHFMELLATLG-VVENCQTLDVID   279
Japanese eel_AT1   GDEVLQ--MLAAVVLAFFLCWVPHQIFHFMHVLALLK-VIENCPTLDIID   279
Zebrafish_AT1b     NG-DEVLNMLAAAALSFFLCWAPHQIFNFMEMLLLLK-VITSCDVVDIID   280
River lamprey_AT1  VLPA-VLAVVLAFLLCWLPYHVLTLLDTLVRLRVLRGCGVTAAVDAAMPV   290
                              *  *

Mudskipper_AT1     TVMPFTICIAYFNSCVNPIVYGFVGRNFRKNLVRLLHCAPASVRGAHPSI   336
Medaka_AT1         TAMPFTICIAYFNSCMNPIVYGFVGRNFRKNLLRLLRCSPGRPAGPHQSI   335
Zebrafish_AT1a     TAMPFTICISYLNSCVNPILYGFVGHNFRKNLLRLLSCG-SGEASNRFSI   328
Japanese eel_AT1   SALPFTICIAYFNSCMNPILYGFVGRNFRRNLLRLLRCG-PGSAPRHSHP   328
Zebrafish_AT1b     TGMPFTICIAFFNSCMNPILYSFVGKNFRRNLLKLLRCS---STSVASHP   327
River lamprey_AT1  AV-----VVAYTNSCLNPLLYSFVGKGFRQGFGRLLRLS-------AYVP   328
                               *** **  * ***  **     **

Mudskipper_AT1     SSKMSAL--SFRASEALSLTVKNKSSSDIK---                    364
Medaka_AT1         SSKMSAL--SFRASEALSLTVKSNVSTDVK---                    363
Zebrafish_AT1a     NSKIDVN--SHCNSGLLNQTSSKNDSASMVKAS                    359
Japanese eel_AT1   SLTTKMSTLSYRASETLRLTSGKAASSQPAK--                    359
Zebrafish_AT1b     ALSTKMSSLSYRTSELSHLSVIKTPSLPRAT--                    358
River lamprey_AT1  RPLASSFTRSSSLR------SRVVEMENIR---                    352
```

65

(c)

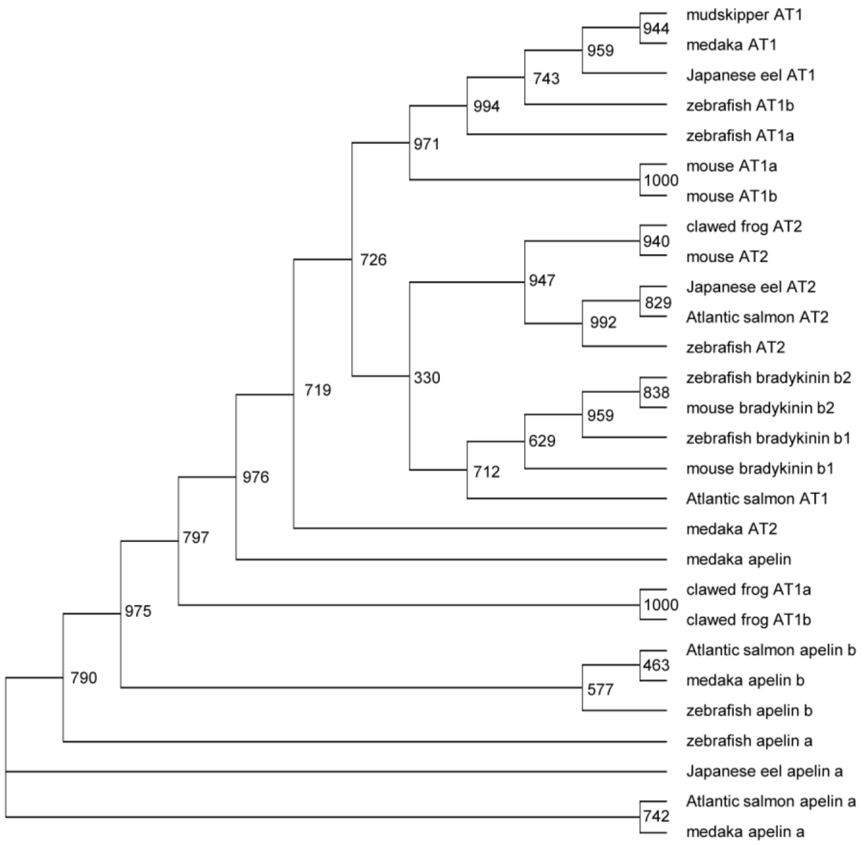

Supplementary Figure 1. Amino acid sequences of angiotensinogen and angiotensin receptor type 1 (AT1). (**a**) Alignments of angiotensinogens of different fish species. Shaded regions indicate conserved amino acids between the mudskipper and other fish in the sequence converted to angiotensin II (AngII). (**b**) Alignments of several AT1s of different fish. (**c**) The phylogenetic tree including AT1, angiotensin receptor type 2 (AT2), apelin receptors, and bradykinin receptors. Numbers represent bootstrap values.

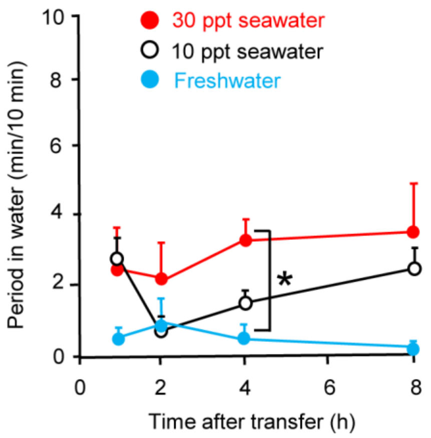

Supplementary Figure 2. Effects of environment salinities on amphibious behaviour.

Mudskippers were transferred from the tank for acclimation (10-ppt seawater) to tanks containing freshwater (●, $n = 6$), 10-ppt seawater (○, $n = 6$) and 30-ppt full-strength seawater (●, $n = 6$). *$P < 0.05$ with two-way repeated measures ANOVA and Dunnett's post-hoc test.

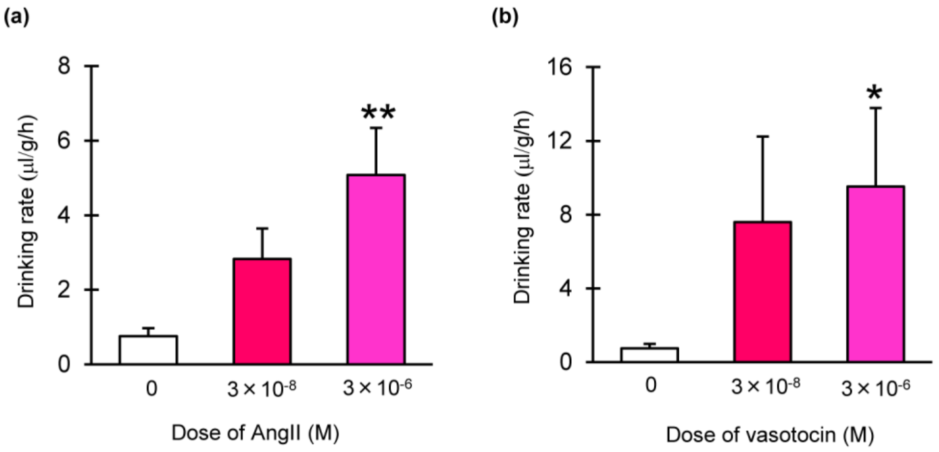

Supplementary Figure 3. Dose-dependent effects of angiotensin II (AngII) and vasotocin on drinking rate. Fish were put in a columnar tank (diameter-65 mm, height-92 mm) containing phenol red in 10-ppt seawater after intracerebroventricular injection with 0.1 μl/g of 3×10^{-6} M (n=5), 3×10^{-8} M (n=5), and vehicle (n = 8) of AngII (**a**) or vasotocin (**b**). Mudskippers were held in water for 1 h. One-way repeated measures ANOVA and Tukey's post-hoc test were used for statistical analysis. Data are shown as mean ± s.e.m. **P* < 0.05, ***P* < 0.01 versus controls.

Supplementary Movie Legends

Supplementary Movie 1. Visualization of swallowing of buccal/opercular water on land by X-ray. In X-ray movies, concentration of shadow indicates the radiopacity of contrast medium. The medium was stored in the esophagus to some extent and immediately flowed to the intestine. The mudskipper does not have a stomach and the esophagus is connected directly to the proximal intestinal swelling. This movie is 5 × faster than actual recording speed.

Supplementary Movie 2. Visualization of the migration to water followed by the capture of water in the buccal/opercular cavity. The contrast medium that the mudskipper acquired in the 96-well microplate hole was held in the buccal/opercular cavity on land (on top of the microplate). This movie is played at actual recorded frame rates.
